# Plants and Natural Products with Activity against Various Types of Coronaviruses: A Review with Focus on SARS-CoV-2

**DOI:** 10.3390/molecules26134099

**Published:** 2021-07-05

**Authors:** Susana A. Llivisaca-Contreras, Jaime Naranjo-Morán, Andrea Pino-Acosta, Luc Pieters, Wim Vanden Berghe, Patricia Manzano, Jeffrey Vargas-Pérez, Fabian León-Tamariz, Juan M. Cevallos-Cevallos

**Affiliations:** 1 Escuela Superior Politécnica del Litoral, ESPOL, Centro de Agua y Desarrollo Sustentable, CADS, Campus Gustavo Galindo Km. 30.5 vía Perimetral, Guayaquil EC090112, Ecuador; susalliv@espol.edu.ec; 2 Escuela Superior Politécnica del Litoral, ESPOL, Centro de Investigaciones Biotecnológicas del Ecuador, CIBE, Campus Gustavo Galindo Km. 30.5 vía Perimetral, Guayaquil EC090112, Ecuador; jaianara@espol.edu.ec (J.N.-M.); pmanzano@espol.edu.ec (P.M.); jefdavar@espol.edu.ec (J.V.-P.); 3 Escuela Superior Politécnica del Litoral, ESPOL, Facultad de Arte, Diseño y Comunicación Audiovisual, FADCOM, Bosque Protector La Prosperina, Campus Gustavo Galindo Km. 30.5 vía Perimetral, Guayaquil EC090112, Ecuador; ypino@espol.edu.ec; 4 Natural Products & Food Research and Analysis (NatuRA), Department of Pharmaceutical Sciences, University of Antwerp, 2610 Antwerp, Belgium; luc.pieters@uantwerpen.be (L.P.); wim.vandenberghe@uantwerpen.be (W.V.B.); 5 Red Universitaria Para la Investigación y Posgrados Vlir Network, Km. 30.5 vía Perimetral, Edificio Principal, Vicerrectorado Académico (2º Piso), Guayaquil EC090112, Ecuador; 6 Escuela Superior Politécnica del Litoral, ESPOL, Facultad de Ciencias de la Vida, FCV, Centro de Investigaciones Biotecnológicas del Ecuador, CIBE, Campus Gustavo Galindo Km. 30.5 vía Perimetral, Guayaquil EC090608, Ecuador; 7 Grupo de Plantas Medicinales y Productos Naturales, Departamento de Biociencias, Facultad de Ciencias Químicas, Universidad de Cuenca, Av. 12 de Abril, Cuenca EC010107, Ecuador

**Keywords:** middle east respiratory syndrome (MERS), severe acute respiratory syndrome coronavirus (SARS-CoV), renin–angiotensin–aldosterone system (RAAS), angiotensin-converting enzyme inhibitors (ACEi), coronavirus disease of 2019 (COVID-19), medicinal plants, antiviral, viral entry inhibitors, biomolecules

## Abstract

COVID-19 is a pandemic disease caused by the SARS-CoV-2 virus, which is potentially fatal for vulnerable individuals. Disease management represents a challenge for many countries, given the shortage of medicines and hospital resources. The objective of this work was to review the medicinal plants, foods and natural products showing scientific evidence for host protection against various types of coronaviruses, with a focus on SARS-CoV-2. Natural products that mitigate the symptoms caused by various coronaviruses are also presented. Particular attention was placed on natural products that stabilize the Renin–Angiotensin–Aldosterone System (RAAS), which has been associated with the entry of the SARS-CoV-2 into human cells.

## 1. Introduction

The Coronavirus Disease 2019 (COVID-19), caused by the Severe Acute Respiratory Syndrome Coronavirus 2 (SARS-CoV-2), was declared a pandemic on 11 March 2020 [1] and is probably the biggest challenge for public health systems in most countries given the limited knowledge about effective treatments [2].

The SARS-CoV-2 belongs to the Coronaviridae family and the Coronavirinae subfamily which has been divided into four genera: α-coronavirus, β-coronavirus, γ-coronavirus and δ-coronavirus [3]. The Human Coronavirus species HCoV (OC43, 229E, NL63 and HKU1), as well as those associated with Severe Acute Respiratory Syndrome (SARS), Middle East Respiratory Syndrome (MERS), and SARS-CoV-2, can cause respiratory tract infection but others such as the species 229E, OC43, HKU1, and NL63 usually cause the common cold [3]. Genetic characterization has shown that SARS-CoV-2 shares almost 80% of the SARS-CoV [4] and 96.2% of the bat β-coronaviruses lineage B [1] genomes. The SARS-CoV-2 belongs to the β-coronavirus group and causes milder symptoms than SARS and MERS but the transmission between people is much faster with an R0 (Basic Reproduction Number) of 3.28 [5] compared to the R0 values around 0.9 for MERS-CoV [2]. The mortality rate for SARS-CoV-2 is 3.4% compared to 9.6% and 35% for SARS-CoV and MERS respectively [6]. The incubation period for SARS is 2 to 10 days, while that of SARS-CoV-2 is 1 to 14 days (Table 1) [4]. Additionally, several studies reported that SARS-CoV-2 and SARS-CoV use the Angiotensin-Converting Enzyme 2 (ACE2) as a receptor to enter target cells, while MERS-CoV uses dipeptidyl peptidase 4 (DPP4) for the same purpose (Table 1) [2]. The alveolar lung and small intestine are potential targets for SARS-CoV-2 due to the high expression of ACE2 [1].

SARS-CoV-2 mainly affects the middle-aged and elderly, as well as people with underlying diseases such as hypertension, diabetes, obesity or with heart and kidney problems, but shows low severity in children [7] although the disease transmission in this age group is still unknown [2] and the infection rates in children are increasing with the emergence of new SARS-CoV-2 variants [9].

Home isolation and quarantine have been applied in most countries to reduce the spread of the disease. However, this measure is also leading to economic, social and political deterioration in the affected countries. Consequently, the cases of anxiety and depression due to confinement as well as the number of deaths due to these causes have increased [10]. The enormous worldwide effort to develop vaccines against COVID-19 is recognized well-known as at least 19 vaccines have entered clinical trials and some vaccines already being applied to people in several countries [11]. However, the rushed development of a vaccine is usually accompanied by numerous challenges including potentially severe side effects and the possible loss of disease protection shortly after vaccination [12]. Moreover, the rise of new virus variants can affect the effectiveness of current treatments.

Similarly, other large-scale trials are in progress for the evaluation of possible therapies, including the World Health Organization (WHO) Solidarity Trial [11]. Pharmaceutical products undergoing clinical trials as potential treatments for COVID-19 include the antiviral nucleotide analog remdesivir, systemic interferons, and monoclonal antibodies [11]. Moreover, the antiparasitic drug ivermectin has been repurposed as a potential antiviral against SARS-CoV-2 and some drugs such as hydroxychloroquine that initially seemed promising have already been discarded by conflicting results through small-scale studies [12].

The accelerated search for a cure involves questions of a bioethical nature which prompts a reflection on the Declaration of Helsinki [2013] as well as the non-maleficence and beneficence principles to enable the use of untested procedures in clinical trials under emergency conditions [13]. It is necessary to implement a sustainable program to improve the health of citizens while a cure for SARS-CoV-2 is developed. Medicinal plants and natural products have the potential for enhancing people’s health and boost the immune system [14]. Plants generally contain a combination of active ingredients or phytochemicals with different properties. Herbal medicinal formulations have been effective in treating emerging and reemerging viral diseases affecting diverse human and animal populations [14]. Plant extracts have shown specific antiviral properties in experimental animal models, which have prompted the formulation of natural products for the treatment of viral diseases [15]. Similarly, the bioactive compounds of medicinal plants can act as immunomodulators and can be combined with other therapies against viral diseases [16]. 

Natural products can help researchers design safe and easily accessible medical treatments [17]. For instance, plants from traditional Chinese medicine (TCM) such as *Scutellaria baicalensis* contain various antiviral compounds, including inhibitors of viral replication [18] and phytochemicals with anti-SARS-CoV-2 potential (Table 2). Furthermore, 125 Chinese herbs were found to contain at least 2 of 13 compounds (betulinic acid, coumaroyltyramine, cryptotanshinone, desmethoxyreserpine, dihomo-γ-linolenic acid, dihydrotanshinone I, kaempferol, lignan, moupinamide, N-cis-feruloyltyramine, quercetin, sugiol, tanshinone IIa) that can inhibit the 3C-Like protease (3CLpro) and Papain-Like protease (PLpro) as well as block the entry, replication and binding of the SARS-CoV-2 Spike protein (S protein) [19]. Similarly, a protective effect against the 229E coronavirus was observed in respiratory cell cultures pre-treated with 50 µg/mL *Echinacea* (Table 2) [20]. In addition, the highly pathogenic SARS and MERS coronaviruses were also inactivated in vitro (IC_50_ 3.2 ug/mL) using the same plant. Other species such as grapefruit (*Citrus* × *paradisi*) have also been used to combat several respiratory infections [21].

The Renin–Angiotensin–Aldosterone System (RAAS) is a cascade of vasoactive peptides that regulate key processes in human physiology. SARS-CoV-1 and SARS-CoV-2 interfere with the RAAS by binding to the Angiotensin-Converting Enzyme 2 (ACE2) which serves as a receptor for both SARS viruses [66]. Overactivation of the RAAS by coronaviruses can contribute to the development of critical symptoms. Several common foods belonging to the families Alliaceae, Apiaceae, Brassicaceae, Cucurbitaceae, Rutaceae, Vitaceae, Zingiberaceae, among others have demonstrated the ability to regulate key RAAS processes [38,60] (Table 3).

Various countries such as Ecuador are considered megadiverse because of the high number of plant species. Various species from megadiverse areas have shown great potential for the treatment of respiratory conditions but have not been tested against coronaviruses (Table 4) [66]. Further research is needed to assess the effect of these species against SARS-CoV-2. The pandemic impact of the 2002 SARS epidemic that began in Foshan, China [38,67], the high mortality rate and the subsequent re-emergence of the disease one year later [60] together with the economic problems caused in Asia encouraged research efforts focused on controlling coronaviruses infections by medicinal plants [68]. The aim of this review was to summarize the available literature on medicinal plants used against various types of coronaviruses, including SARS CoV-2 [67]. Special emphasis was placed on species located in Ecuador as one of the megadiverse countries.

## 2. Methods

### Literature Search

The PubMed, NCBI, Elsevier databases were used for searching natural compounds and medicinal plants with pharmacological activity against the SARS, MERS or SARS-CoV coronaviruses. Keywords like coronavirus; COVID-19; medicinal plants; active principle; natural compounds; inhibitor; SARS; MERS or SARS-CoV-2; Spike protein; RAAS; Angiotensin-Converting-Enzyme Inhibitors (ACEi); Angiotensin Receptor Blocker (ARB) were used to carry out the search. In addition, studies published since 2002 were reviewed, as this was the year in which SARS was reported for the first time [73]. The common name of the studied plants was determined with the help of an expert botanist, using the references “Plantas Útiles de Litoral Ecuatoriano de Flor María Valverde Vadillo” [74] and the “Enciclopedia de las Plantas Útiles del Ecuador”, and the databases “Herbario Rapid Reference” (https://plantidtools.fieldmuseum.org/es/rrc/5581) Date accessed: 19 April 2019 and “Trópicos” (https://www.tropicos.org/home) Date accessed: 14 May 2019.

## 3. Pathogenesis of SARS-CoV-2

**Figure 1:**
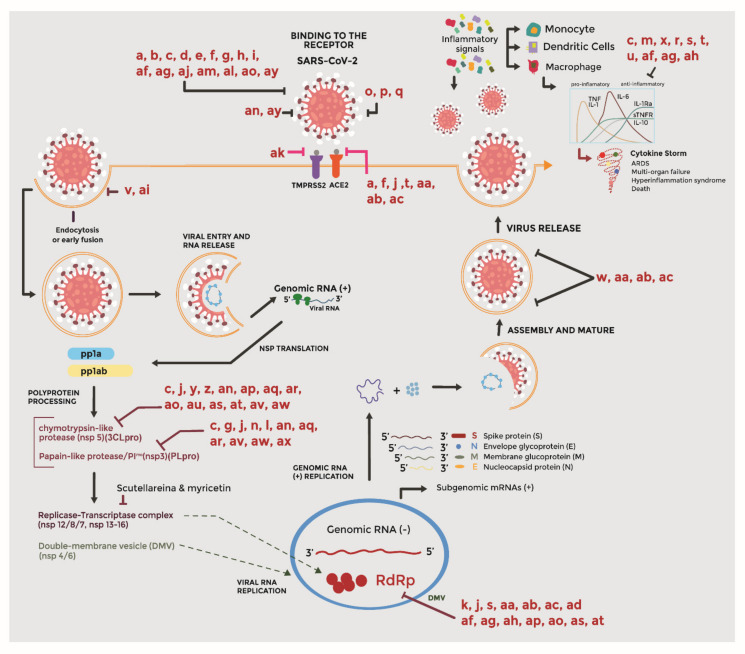
Various active principles and their mechanism of action. The infection cycle of SARS-CoV-2 in human cells. The SARS-CoV-2 spike (S) protein binds to ACE2 in host cells followed by priming of protein S by transmembrane protease serine 2 protease (TMPRSS2). Then, the virus produces the polyproteins pp1a and pp1ab, which are processed by viral proteases (3CLpro/Mpro, PLpro) to non-structural proteins (nsps), including RNA-dependent RNA polymerase (RdRp). Viral RdRp synthesizes a full-length complementary negative-strand RNA as a template for the production of the positive strand genome of the virus. Subgenomic mRNAs are then translated into structural proteins in the rough endoplasmic reticulum or in the cytosol. The viral genomic RNA is encapsulated by the nucleocapsid protein N and, finally, the virus is released by exocytosis. The blunt arrows indicate the possible targets of the active principles of medicinal plants. Irreversibly interference with viral docking receptors: Caphtharic acid (**o**), cichoric acid and echinacoside from *Echinacea purpurea* (**p**), vitamins D, C and Zn (**q**). Entry locks: Emodin (**a**), lectins (**b**), quercetin (**c**), catechin (**d**), naringenin (**e**), hesperetin (**f**), baicalin (**g**), epigallocatechin (**h**), gallocatechin gallate (**i**), prodelphinidin (**af**), gallocatechin (**ag**), saikosaponins derivatives of oleanane from *Heteromorpha arborescens* and *Bupleurum* spp. (**aj**), glycyrrhizine (**al**), Licorice (**am**), desmethoxyreserpine (**ao**), dihydrotanshinone I (**ay**). ACE2 receptor blocking: Emodin (**a**), hesperetin (**f**), kaempferol (**j**), anthocyanins (**t**), phenolic compounds: tannic acid (**aa**), 3-isotheaflavin-3-gallate (**ab**) and theaflavin-3,3′-digallate (**ac**) from *Camellia sinensis*. TMPRSS2 receptor blocking: [6]-gingerol (**ak**). Block the entry of cells through endocytosis: Butanol extract (**v**) and procyanidins (**ai**) from *Cinnamomum verum*. Inhibit 3CLpro: Quercetin (**c**), kaempferol (**j**), curcumin (**y**), sinigrin (**z**)**,** eugenol (**an**), betulinic acid (**ap**), coumaroyltyramine (**aq**), cryptotanshinone (**ar**), desmethoxyreserpine (**ao**), Dihomo-γ-linolenic acid (**au**), lignan (**as**), sugiol (**at**), N-cis-feruloyltyramine (**av**), Tanshinone IIa (**aw**). Inhibit PLpro: Quercetin (**c**), baicalin (**g**), kaempferol (**j**), myricetin (**n**), scutellarein (**l**), eugenol (**an**), coumaroyltyramine (**aq**), cryptotanshinone (**ar**), N-cis-feruloyltyramine (**av**), Tanshinone IIa (**aw**), moupinamide (**ax**). Affinity with S protein: Eugenol (**an**), dihydrotanshinone I (**ay**). Viral replication: Aescin (**k**), kaempferol (**j**), resveratrol (**s**), prodelphinidin (**af**), gallocatechin (**ag**), epigallocatechin isomer (**ah**) from *Pelargonium sidoides*, essential oils: β-ocimene, 1,8-cineole, α-pinene and β-pinene (**ad**), phenolic compounds: tannic acid (**aa**), 3-isotheaflavin-3-gallate (**ab**) and theaflavin-3,3′-digallate (**ac**), betulinic acid (**ap**), desmethoxyreserpine (**ao**), lignan (**as**), sugiol (**at**). Affects the release or assembly of the virus: Phenolic compounds: tannic acid (**aa**), 3-isotheaflavin-3-galalate (**ab**) and theaflavin-3,3′-digallate (**ac**), lectin agglutinin (**w**) from *Hippeastrum striatum*. TNF-β, IL-1β expressions: Quercetin (**c**), luteoloside (**m**), chlorogenic acid (**x**) geranylated flavonoids (tomebrin A, B, D and E) (**r**), resveratrol (**s**), anthocyanins (**t**), gallic acid (**u**), prodelphinidin (**af**), gallocatechin (**ag**), epigallocatechin isomer (**ah**).

SARS-CoV-2 relies on its S protein to attach to human cells having an ACE2 receptor. Studies have shown that SARS-CoV-2 has a higher ACE2 binding affinity than SARS-CoV, supporting an efficient cell entry [75]. The S protein from SARS-CoV-2 consists of subunits S1 and S2. While S1 is important for the virus attachment to the ACE2 receptor, S2 allows the fusion of the virus and cell membranes followed by the internalization of the viral genetic material. Therefore, after attachment to the ACE2 receptor, the S protein needs to be primed at the S1–S2 site by cellular proteases such as the Transmembrane Serine protease 2 (TMPRSS2) [35]. Therefore, the virus is capable of infecting human cells containing both ACE2 receptors and proteases such as the TMPRSS2, including lungs, small intestine, heart and kidney cells, as well as the nose, nasopharynx and oral mucosa [35]. Once inside the cell, the viral genetic material undergoes replication, synthesis of the S protein as well as other polyproteins. Figure 1 shows the infection process of SARS-CoV-2 in human cells. 

The synthetized polyproteins are then processed by a 3C-like protease (3CLpro) also known as the main protease (MPro) and a PLpro to produce 16 nonstructural proteins (Nsp), including the Nsp13 helicase, responsible for the replication and transcription of the viral genome [18]. After cell entry and multiplication, the virus can cause inflammatory responses in the host attributed to an excessive release of cytokines. This cytokine storm has been associated with severe damage to the lungs, blood hypercoagulation, cardiac arrest and lymphocytopenia among other life-threatening conditions [76].

### 3.1. The Renin–Angiotensin–Aldosterone System as Affected by SARS-CoV-2

During the SARS-CoV-2 infection, the virus sequesters ACE2 causing the instability of the RAAS and contributing to various symptoms of COVID-19. 

**Figure 2:**
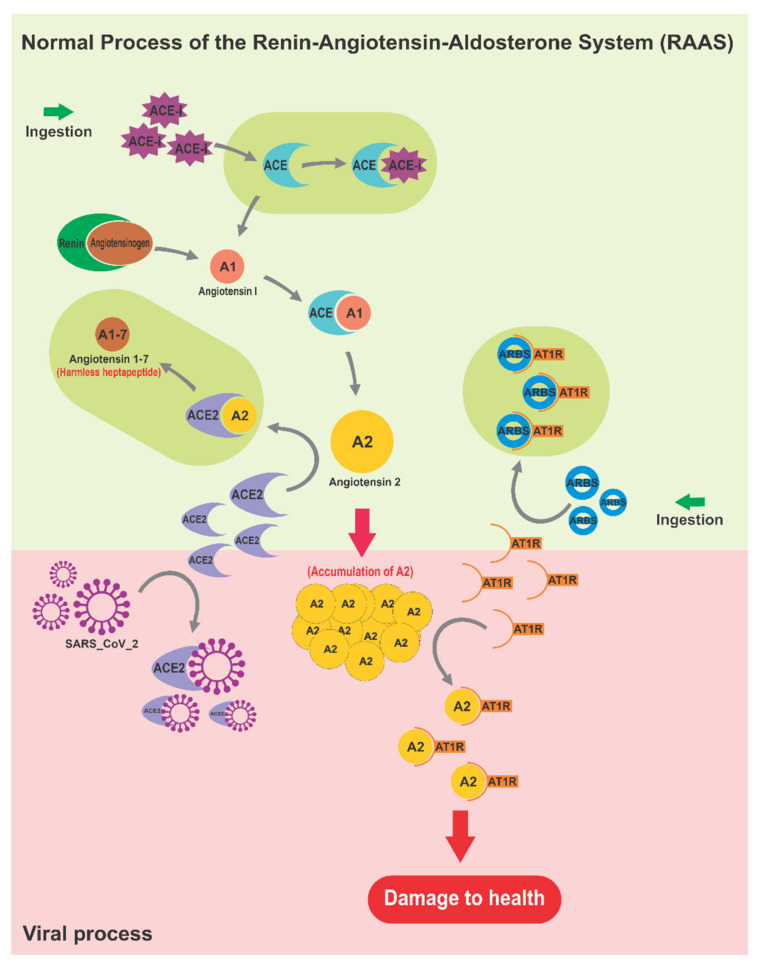
Mechanism of action of SARS-CoV-2 on the Renin–Angiotensin–Aldosterone System (RAAS) and its possible regulation by the Angiotensin converting enzyme inhibitors (ACEi), Angiotensin receptor blockers ARBs or Angiotensin converting enzyme (ACE2) that converts AI to A1-7 to restore the RAAS.

A stressed organism is usually more predisposed to infections by microorganisms [73]. Frequent or very strong episodes of stress caused by an overactivated RAAS include an excessive conversion of Angiotensin I (AI) into Angiotensin II (AII) by the ACE [77]. AII binds to the Angiotensin II Type I Receptor (AT1R), causing instability of blood pressure [78] as well as cardiovascular, renal [79] and prothrombotic issues [80]; myocardial dysfunction [81]; altered activity of the sympathetic nervous system [82]; and chronic hypertension in obese individuals [83]. AII is considered a cytokine with pro-inflammatory properties and the accumulation of this molecule can induce chemotaxis, contributing to a storm of cytokines [81,84]. To regulate the over-activated RAAS, ACE2 inactivates AII generating the harmless heptapeptide Angiotensin 1–7 (A1-7) with a powerful vasodilator function [85]. However, SARS-CoV-2 disrupts this mechanism after hijacking ACE2, causing the accumulation of AII and contributing to various symptoms of COVID-19 [66]. Therefore, the over-activation of RAAS should be prevented to reduce the severity of the infection [86]. Specific foods and plants that modulate the RAAS [60] can prevent the coronavirus entry or alleviate the COVID-19 symptoms. Figure 2 shows the effect of SARS-CoV-2 on the RAAS.

### 3.2. Immune System Boosting Plants and Foods

At present, different herbal plants are being subjected to studies on their ability to strengthen the immune system and cope up with the virus and some phytocompounds have already shown potential to mitigate the incidence of infection [87]. For instance, various plant polyphenols can initiate a cellular accumulation to then trigger signaling pathways and immune responses to infection. In addition, polyphenols are potent inhibitors of the COVID-19 protease (Mpro) [87]. 

Natural polysaccharides and terpenoids are immunomodulatory as well as adaptogenic compounds and are also recognized for their antiviral, immunomodulatory, antitumor and anticoagulant bioactivities. Similarly, giloy herbs can stimulate IgG antibody response, macrophage activation, induction of cell-regulated immunity, and humoral immunity [87]. Moreover, several plant triterpenes such as dammaradienol, dammarenediol-II, hydroxyhopanone. dammarenolic acid, hydroxymarenone-I, ursonic acid, shoic acid, eichlerianic acid and hydroxyoleanonic lactone [87] play a vital role in the modulation of cellular metabolism [88].

Sulfated polysaccharides are a structurally multifaceted class of biomolecules with diverse physicochemical characteristics well recognized in the field of medicine and pharmaceutical sciences [29]. They have immunomodulatory properties and bioactivities [89]. Furthermore, they are selective inhibitors or suppressors of enveloped viruses, e.g., HSV, HIV, human cytomegalovirus, respiratory syncytial virus, and influenza [89].

The biomolecules hispidin, lepidine E, and folic acid from *Citrus* sp. inhibit the 3CL hydrolase enzyme known to counteract the host’s innate immune response [90]. Similarly, Benzene 123 Triol from *Nilavembu kudineer* has shown immunomodulatory activity [91] while *Exocarpium Citri grandis* (Flavonoids and Naringin) stimulated the antiviral immune response and showed antitussive, expectorant and helped relieve pulmonary fibrosis [89]. Moreover, *Allium sativum* (Allicin) stimulated the activity of immune cells and inhibited the release of pro-inflammatory cytokines dependent on Necrosis Tumoral Factor alfa (TNFα) as well as the migration of neutrophilic granulocytes, a crucial process during inflammation [46]. The plant species *Acacia senegal*, *Laportea aestuans*, and *Citrus* spp (Hesperidin) increased antioxidant defenses, modulated the activity of the immune system, and eliminated reactive oxygen species. In addition, *Curcuma longa* (Curcumin) also enhanced immunity [46].

Foods containing curcumin, allicin, papain, ginsenoside, mangoosteen, chloroquine, etc., have shown a direct effect on dendritic cells, natural killer cells (NK), lymphocytes and antibodies to protect the human body from foreign particles [89].

## 4. Bioactive Compounds in the Mechanisms of the Virus–Host Interaction

Table 2 shows the plant species with activity against various coronaviruses. 

### 4.1. Entry Inhibitors 

Many plant bioactive compounds typically prevent the entrance of the viral particle into the host cell [87]. SARS-CoV entry inhibitors are divided into two categories: the first consists of molecules that bind to the ACE2 and TMPRSS2 receptors while the second comprises compounds that bind to the virus and prevent interaction with the cell receptors and membrane fusion [92]. The molecule [6] gingerol from *Zingiber officinale* inhibits the growth of the coronavirus by blocking the cell’s TMPRSS2 receptor [21].

The TCM’s Jinchai consists of plant species such as *Lonicera japonica* and *Bupleurum chinense* among others, that prevent the coronavirus entry into cells and inhibit general viral replication as well as the specific 3CLpro-mediated replication [29]. One of the main active components of Jinchai is baicalin which inhibited antiviral activity with an Effective Concentration (EC_50_) of 12-50 µg/mL in SARS-CoV-infected fetal rhesus monkey kidney cell line (fRHK4) and EC_50_ of 100 µg/mL in Vero-E6 cells [93].

Flavonoids stand out among the blockers of the ACE2 receptor, but they have also shown anti-replication activities. Similarly, compounds such as baicalin, epigallocatechin gallate, gallocatechin gallate, derivatives of kaempferol, myricetin, quercetin and scutellarein are other major constituents of TCM used to treat SARS by inhibiting the entry and replication of the virus [64]. 

The flavonoid hesperetin has the potential to inhibit ACE2 and block SARS-CoV-2 infection by binding to viralS protein, helicase, and protease sites of the ACE2 receptor [29].

Alternatively, computational analysis revealed that hesperidin, baicalin and kaempferol 3-O-rutinoside can block SARS-CoV-2 infection by weakening the adsorption of virus to cells [19,46]. Similarly, procyanidins and the butanol extract of *Cinnamomi Cortex* (bark of *Cinnamomum verum*) have shown antiviral effects at the RNA level, in addition to inhibiting SARS-CoV infection with an IC_50_ of 29.9 ± 3.3 μM (Table 2 and Table 3) [30]. Additionally, cinnamon extract inhibited wild-type SARS-CoV infection in vitro with an IC_50_ of 43 μM and blockage of the virus entry to the cell was suggested as the possible mechanism of action [32]. The polyphenol epigallocatechin gallate (EGCG) from *Camellia sinensis* (green tea) inhibited the spread of the bovine coronavirus and interfered with the viral adsorption to bovine kidney cells [94]. 

Among the virus-binding molecules, lectins have emerged as a new class of antivirals thanks to their ability to bind to the glycosylated molecules found on the surface of viruses such as the SARS-CoV spike glycoprotein [24]. One of the most potent molecules reported against SARS-CoV is the mannose-binding lectin isolated from leek (*Allium porrum* L.), with an EC_50_ of 0.45 μg/mL and a selectivity index >222 (Table 2 and Table 3) [29]. Specific N-acetylglucosamine lectins obtained from tobacco (*Nicotiana tabacum* L.) and stinging nettle (*Urtica dioica* L.) were also active against SARS-CoV with selectivity indexes of >77 and >59, respectively [24]. Additionally, the mannose-specific lectin from *Hippeastrum striatum* (Lam.) has the potential to inhibit the final step of the virus infection cycle [24,87]. Similarly, triterpenoids such as glycyrrhizin from the licorice plant, *Glycyrrhiza glabra* L., have been reported to have in vitro anti-SARS effects with an EC_50_ of 300 µg/mL [51]. These natural compounds interfere with virus–host fusion steps through the envelope of the predominant heptad repeat 2 domains in viral envelopes [89].

Emodin is a natural anthraquinone derivative and an active ingredient of medicinal plants such as rhubarb (Genus *Rheum*) (Table 2 and Table 3), *Polygonum cuspidatum*, *Aloe vera*, *Senna obtusifolia* [59] and *Cassia tora* L [28]. Emodin blocked SARS-CoV entry to host cells by binding to the S proteins and interfering with the 3CLpro activity of the virus, thus preventing the formation of the Nsp required for replication [27]. In trials involving SARS-CoV and OC43, emodin significantly blocked, in a dose-dependent manner, the interaction between SARS-CoV S protein and ACE2, inhibited the ion channel 3a and interrupted the release of new coronaviruses [22]. Similarly, terpenoids from medicinal plants exhibit general antiviral effects in vitro against SARS-CoV [29]. Oleanane-type saikosaponins found in medicinal plants such as *Bupleurum* spp. and *Heteromorpha* spp. prevented the entry of SARS-CoV into the cell [46].

### 4.2. Protease Inhibitors

Proteases are key players in the pathogenesis caused by SARS-CoV and SARS-CoV-2 as they are involved in the S protein activation and viral replication. Therefore, protease inhibitors can aid the COVID-19 treatment. Because of the good binding affinity for Mpro and S protein of eugenol and curcumin, these compounds can be considered promising anti-SARS-CoV agents [22,95]. Curcumin inhibited SARS-CoV 3CLpro with an IC_50_ value of 23.5 μM [22,92]. Similarly, various phenolic tea constituents, such as tannic acid, 3-isotheaflavin-3-gallate and theaflavin-3,3-digallate (Table 2 and Table 3) also inhibit SARS-CoV 3CLpro with IC_50_ values between 3, 7 and 9.5 μM, respectively [37]. Similarly, a cell-based study showed that sinigrin significantly blocked the cleavage process of 3CLpro with an IC_50_ of 752 μM. Sinigrin is a glucosinolate found in some plants of the Brassicaceae family, such as Brussels sprouts, broccoli, and black mustard seeds [29] (Table 2 and Table 3).

*Scutellaria baicalensis* polysaccharides, polyphenols and polyglycans can inhibit immune regulation and have shown antioxidant and antiviral activity [57]. The flavonoids scutellarein and baicalin from the same species inhibited SARS-CoV Nsp13 helicase [56], while myricetin reached an IC_50_ of 2.71 μM against the virus [61]. These two compounds potently inhibited Nsp13 in vitro by affecting the ATPase activity of SARS-CoV [57].

### 4.3. Replication Inhibitors

Inhibitors of viral replication are amongst the key molecules to fight coronavirus diseases. The phenolic compounds from *Melia azedarach* (cinamomo or chinaberry tree) and *Camellia sinensis* (green tea) have shown antiviral activity due to the inhibition of RNA polymerase or the RNA-dependent proteases involved in the replication of the coronavirus RNA [29]. Additionally, tea extracts can also affect the virus assembly and release [96]. Similarly, the consecutive application of stilbene derivatives such as resveratrol at 62.5 μM partially mitigated MERS-CoV-induced cell death and reduced the replication of infectious MERS-CoV by 10-fold [63]. Similarly, concentrations below 0.5 mg/mL of stilbene derivatives like resveratrol inhibited the replication of SARS-CoV in vitro [62]. These compounds are found in different plants, including the *Vitis vinifera* L. grape and berries of the genus *Vaccinium* (Table 2 and Table 3) [30]. Compounds in berries have been suggested to block the virus entry to cells through endocytosis [97].

In general, natural flavonoids such as quercetin, catechin, naringenin and hesperetin are the most abundant polyphenols in the human diet, as they are found in fruits and vegetables as glycosides or acylglycosides [95]. Naringenin exhibited a partial inhibition of SARS-CoV-2 replication observed at 24 h post-infection (hpi) in cells upon Two-pore channel 2 (TPC2) silencing while stronger inhibition was observed at 48 and 72 hpi [36]. 

The standardized extract of *Pelargonium sidoides* (EPS 7630), mainly containing polyphenolic compounds such as prodelphinidin, gallocatechin and its stereoisomer epigallocatechin [53,98], is an approved treatment for acute bronchitis in Germany and other countries [53]. Concentrations up to 100 μg/mL of EPS 7630 interfered with the replication of human coronavirus as well as the seasonal influenza A virus Hemagglutinin Type 1 and Neuraminidase Type 1 (H1N1, H3N2), respiratory syncytial virus, parainfluenza virus and coxsackie virus [52] and inhibited the entry and replication of 229E with EC_50_ of 44.50 ± 15.84 μg/mL [99].

The essential oils of *Laurus nobilis* and *Salvia officinalis* have also shown significant anti-replication activity against SARS-CoV with an Inhibitory Concentration (IC_50_) value of 120 μg/mL [58]. Similarly, the essential oils from *Thuja orientalis* (β-ocimene, 1,8-cineole, α-pinene and β-pinene) also inhibited SARS-CoV replication [58] and the aescin isolated from the horse chestnut tree also inhibited SARS-CoV replication at non-toxic concentrations [22,100].

### 4.4. Virucidal Activity

The inactivation of the viral particles is another strategy to combat respiratory diseases. *Echinacea purpurea* extracts available as the commercial product Echinaforce^®^ showed dose-dependent inhibition of 229E infectivity in respiratory epithelial cells and this extract irreversibly inactivated the virus with an IC_50_ of 3.2 μg/mL [101] and 9 ± 3 μg/mL in another study [76]. The multicomponent extract non-specifically and irreversibly interfered with viral docking receptors to block the infectivity of pathogens [102]. Similarly, inhibition for MERS-CoV was observed with 10 μg/mL of Echinaforce^®^, reducing viral infectivity by 99.9% at 50 μg/mL [41]. Combining *E. purpurea* with vitamin D, vitamin C, and zinc has been suggested to reduce the risk of infection and death from SARS-CoV-2 [103]. A scientific review concluded that along with vitamin D, vitamin C and zinc, *Echinacea* extracts are pivotal in terms of prevention and treatment (shortening the duration and/or lessening the severity of symptoms) of common colds [104].

### 4.5. Immunomodulatory Agents

Generally, the viral loads observed in patients correlate with the severity of symptoms and mortality. The multisystem inflammatory syndrome, known as cytokine storm, occurring in many COVID-19 patients, is caused by an uncontrolled replication of the virus resulting in an over-activation of the immune system, including high levels of pro-inflammatory cytokines, i.e., interleukin-1β (IL-1β) and TNFα [105]. The geranylated flavonoid tomentin E from *Paulownia tomentosa* inhibited SARS-CoV (PLpro) in a dose-dependent manner with an IC_50_ between 5.0 and 14.4 μM and reduced the concentration of the pro-inflammatory cytokines IL-1β and TNFα [51]. Similarly, one study observed that chlorogenic acid, luteoloside, quercetin, and other compounds in *L. japonica*, exhibited anti-inflammatory, antiviral, antibacterial, and antioxidant activity and enhanced immune response. It is known that one of the main possible anti-SARS mechanisms is decreasing the expression of inflammatory mediators such as the transforming growth factor-beta (TNF-β) and IL-1β [49].

Anthocyanins are found in red to violet fruits such as berries of the genus *Vaccinium*, blackberry, among others (Table 2 and Table 3) [106]. Anthocyanin metabolites, such as the protocatechuic acid, were shown to weakly inhibit Nitric Oxide (NO) production and TNF-α secretion in Lipopolysaccharide-Gamma interferon-induced macrophages (LPS-INF-γ) [107]. Additionally, gallic acid decreased the secretion of the inflammatory mediators monocyte chemoattractant protein 1 (MCP-1), intercellular adhesion molecule 1 (ICAM-1), and vascular cell adhesion molecule 1 (VCAM-1) in endothelial cells [83]. However, the anthocyanins concentrations used for the anti-inflammatory activity tests cannot be achieved physiologically [107]. Similarly, *Echinacea* has also been proposed as a suppressor of the immunoinflammatory cascades observed in COVID-19, thanks to the plant’s ability to activate the anti-inflammatory cannabinoid-2 (CB2) receptors and peroxisome proliferator-activated receptors gamma (PPARγ) [102].

### 4.6. Regulators of RAAS

Table 3 summarizes medicinal plants and purified bioactives with potential benefits against SARS-CoV-2, especially those that modulate the RAAS. For example, the ethanolic extract of *Thymus vulgaris* (thyme), among other plants has shown the inhibitory capacity of AT1R [34]. 

The onions tunic extract, rich in flavonols like quercetin, has been shown to be a competitive inhibitor of ACE, comparable to pure quercetin (IC_50_: 0.36 ± 0.04 and 0.34 ± 0.03 μg/mL respectively). This same extract further revealed competitive ACE inhibition with the substrate, N-[3-(2-furyl) acryloyl]-L-phenylalanylglycylglycine [26]. Agrawal and collegeus reported that quercetin can interfere with various stages of the coronavirus entry and replication cycle, such as PLpro, 3CLpro and nucleoside-triphosphatase (NTPase)/helicase, showing pleiotropic activities and lack of systemic toxicity [25]. Similarly, EGCG also inhibited ACE and blocked the AII binding to AT1R in vitro, showing the potential to control the symptoms of various diseases, especially those of a respiratory nature [108]. Further research is needed to assess the potential of EGCG for the treatment of symptoms caused by coronaviruses.

### 4.7. Unknown Mechanisms of Action

The lycorine purified from *Lycoris* spp. was identified as a promising anti-SARS-CoV bioactive compound with an EC_50_ value of 34.5 ± 2.6 μg/mL, by poorly understood mechanisms [50]. Flavonoids, benzofurans, stilbene, polyhydroxylated alkaloids, and kuwanons from *Morus* spp. have shown a large variety of pharmacological activities including antiviral activity but the mechanism is also unclear [68,109]. The same is through for the compounds from *Ginkgo biloba* (ginkgolide, terpenic lactones, flavonoids, polyphenols, oleic acid, among others) [110]. Therefore, further research is needed to resolve their antiviral mechanism(s) of action.

Recently, two naturally occurring alkaloid-derived compounds (homoharringtonine and emetine), effectively inhibited the SARS-CoV-2 in Vero E6 cells with an estimated EC50 of 2.55 μM and 0.46 μM, respectively [111]. Similarly, emetine has been reported as an inhibitor of hCoV-OC43, hCoV-NL43, SARS-CoV MERS-CoV and MHV-A59 in vitro with EC50 at the low micromolar range. However, the study did not disclose the mechanisms by which both compounds induced anti-SARS-CoV-2 activity [112]. Emetine is a natural alkaloid isolated from Psychotria ipecacuanha and belongs to the methine class of alkaloids [113]. Similarly, homoharringtonine is a natural alkaloid derived from some species of the genus Cephalotaxus. This drug is a protein synthesis inhibitor and has been approved by the Food and Drug Administration (FDA) to treat chronic myeloid leukemia [112].

## 5. Risks Associated with the Incorrect Use of Natural Products

Although many of the plant species hold promise to reduce or mitigate COVID-19 symptoms, it is necessary to further validate their potential health benefits with clinical trials as well as to identify potential side effects. Despite the reported health benefits, high doses of ginkgo (*Ginkgo biloba*) [Table 2] can cause an increase in cerebral blood flow, and affect people with peptic ulcer and coagulation disorders [114,115].

Although no adverse effects have been reported in the consumption of ginger ([6]-gingerol) (Table 2), irritation of the gastric mucosa has sometimes been mentioned. Similarly, turmeric should not be used in case of infections or inflammation of the hepato-bile duct or jaundice [115] and only the stem of rhubarb (Table 3) can be ingested as the leaves contain a large amount of oxalic acid that causes kidney stones [30]. Moreover, the excessive use of *Aloe* species (Table 2) can cause damage to the epithelium and the intestinal mucosa, hemorrhagic diarrhea, and kidney damage. Doses greater than 1 g/day are not recommended for pregnant women, women during menstruating periods or people suffering from kidney disease [115]. Consequently, medical observation is recommended for people who have never consumed any of the plants mentioned in this work. People must be properly informed of the contraindications before combining medicinal plants with any treatment against the symptoms of COVID-19 [116] in order to avoid a counterproductive effect.

## 6. Conclusions

Scientific evidence of medicinal plants and foods that can help to mitigate the symptoms of COVID-19 has been growing since the start of the pandemic. Therefore, it is important to promote the consumption of natural products under the supervision of experts in the medical, nutritional and pharmaceutical areas as well as encouraging the generation of scientific information that promotes the manufacture of plant-based products that help to better protect the people against the SARS-CoV2.

The identification of the antiviral mechanisms of natural agents acting in different stages of the viral life cycle offers hope for future antiviral therapies [16]. In addition, the elucidation of the mechanism of action of natural compounds against COVID-19 will contribute to discover promising anti-COVID-19 natural drugs [108]. However, it is important to emphasize that medicines for the treatment of COVID-19 should not be replaced by untested natural products. Good practices of bioprospecting of medicinal plants should be fostered, in order to increase the interests of the ancestral people from developing countries [116]. This will allow the promotion of the development of new natural products that mitigate the symptoms of this COVID-19 without leaving the vital specialized medical treatment [32].

## Figures and Tables

**Table 1:** Pathogenetic and epidemiological characteristics of SARS-CoV-2, SARS-CoV and MERS-CoV.

Species	Receptor	Incubation Period	RO	Case Fatality Rate	References
SARS-CoV-2	ACE2	1 to 14 days	3.28	3.4	[3,5,7]
SARS-CoV	ACE2	2 to 10 days	1.7–1.9	9.6	[4,8]
MERS-CoV	DPP4		0.9	35	[2]

**Table 2:** Medicinal plants and natural products with inhibitory activity against various types of coronaviruses.

Scientific/Common Name	Active Principle	Virus/ Antiviral Activity	Reference
*Aesculus hippocastanum* CN: Horse-chestnut	Aescin (k)	SARS-CoV/Inhibits viral replication	[22]
*Allium ampeloprasum* Var. porrum J. Gay CN: Leek	Mannose-binding specific lectin (b)	SARS-CoV/Ability to bind to the glycosylated molecules found on the surface of viruses, including the spike glycoprotein	[23] [24]
*Allium cepa* L. CN: Onion	Flavonols: quercetin, quercetinglycosides (isoquercitrin, quercitrin and rutin) (c) and kaempferol (j)	SARS-CoV2/Interfere with various stages of the coronavirus entry and replication cycle such as PLpro, 3CLpro, and NTPase/helicase; Inhibits ACE by competing with the substrate, N-[3-(2-furyl) acryloyl]-L-phenylalanylglycylglycine	[25] [26]
*Brassica oleracea* L. CN: Broccoli	Glucosinolate type sinigrin (z)	SARS-CoV/blocks the cleavage process of 3CLpro	[27] [28]
*Bupleurum* spp. CN: Bupleurum	Oleanane-type saikosaponins (aj)	SARS-CoV/Inhibit human coronavirus entry into cells, general replication, and specific 3CLpro mediated replication	[29]
*Cassia tora* L. CN:	Anthraquinone derived emodin (a)	Inhibitory activities on angiotensin-converting enzyme.	[28]
*Cinnamomumverum* J. Presl CN: Cinnamon (cortex)	Butanol (v), procyanidins (ai)	SARS-CoV/Possibly blocks the entry of cells through endocytosis	[30] [31] [32]
*Curcuma* spp. CN: Turmeric	Curcumin (y), Eugenol (an)	SARS-CoV/Inhibits 3CLpro (y); Good binding affinity with Mpro and S protein (an)	[33] [34]
*Citrus* spp. CN: Three main species in the country: Citrus maxima (Rumph. ex Burm.) Merr; Citrus medica L.; Citrus reticulata Blanco.	Hesperetin (f) and naringenin (e)	SARS-CoV-2/(f) Inhibits ACE2 and inhibit the entry of virus into cells host by binding to S protein, helicase, and protease sites on the ACE receptor HCoV229E/(e) Partial inhibition of 229E replication in cells silenced for TPC2 by siRNA	[35] [36]
*Camellia sinensis* Kuntze CN: Green tea	Phenolic compounds: Tannic acid (aa), 3-isotheaflavin-3-galalate (ab) and theaflavin-3,3′-digallate (ac)	Coronavirus in general/Possibly inhibition of RNA polymerase or RNA-dependent proteases; They can also affect the release or assembly of the virus; inhibits ECA and blocking AII receptor binding in vitro, avoiding symptoms of various diseases, especially those of a respiratory nature	[37] [38]
*Melia azedarach* L. CN: Cinamomo			[39] [37]
*Echinacea purpurea* Moench CN: Echinaceae^®^	Caphtharic acid (o), cichoric acid (p) and echinacoside (p)	MERS-CoV, 229E/The extract non-specifically and irreversibly interferes with viral docking receptors (eg, influenza) to block infectivity of pathogens	[40] [41]
*Ginkgo biloba* L. CN: Ginkgo	Ginkgolide, terpenic lactones, flavonoids, polyphenols, oleic acid, among others.	SARS-CoV/Antiviral mechanism is unclear	[19] [42]
*Glycyrrhiza glabra* L. CN: Licorice (root)	Licorice (am) y glycyrrhizin (al)	SARS-CoV/Modulate some virus-host fusion functions through the envelope of the repetition domain 2 of the predominant heptad in viral envelopes; Improvement of the function of upper respiratory mucosal immune system; Inhibit viral adsorption and penetration	[29] [22] [43] [44]
*Heteromorpha arborescens* Cham. CN: Parsley tree	Oleanane-type saikosaponins (aj)	SARS-CoV/Prevent the entry of SARS-CoV into the cell	[45] [46]
*Hippeastrum striatum* Lam CN: Lily	Lectin agglutinin (w)	SARS-CoV/Inhibits the end of the virus cycle infection	[29] [47]
*Lonicera japonica* Thunb CN: Madreselva *Eriobotrya japonica* Thunb CN: Níspero	Quercetin (c), luteoloside (m), chlorogenic acid (x)	SARS-CoV, RSV, HIV, HSV, PRV and NDV/This mechanism possibly is due to diminishing the inflammation mediators and TNF-β, IL-1β expression. Anti-inflammatory, antiviral, antibacterial, antioxidant activity. Enhances the immune response.	[48] [49]
*Lycoris* spp. CN: hurricane lilies or cluster amaryllis	Lycorine	SARS-CoV/Compound with extensive antiviral activities. However, the antiviral mechanism of this molecule is unclear	[50]
*Morus alba* L. CN: Tree mulberry	Aliphatic, aromatic phenolic, heterocyclic and aliphatic cyclic compounds	SARS-CoV and MERS-CoV/Antiviral mechanism is unclear	[19] [42]
*Nicotiana tabacum* L. CN: Tobacco	N-acetylglucosamine specific lectins (b)	SARS-CoV/Ability to bind to the glycosylated molecules found on the surface of viruses, including the spike glycoprotein.	[23] [29]
*Paulownia tomentosa* Steud CN: Kiri	Flavonoids: (quercetin (c), catechin (d) and naringenin (e) and geranilated flavonoids (tomentin A, tomentin B, tomentin C, tomentin D, tomentin E) (r)	SARS-CoV/Inhibits SARS-CoV (PLpro) by reducing the concentration of pro-inflammatory cytokines (IL-1β) and TNFα	[51]
*Pelargonium sidoides* D.C. CN: Geranium	Prodelphinidin (af), gallocatechin (ag) and their epigallocatechin stereoisomer (ah)	H1N1, H3N2, HCoV-229E/inhibits the entry and replication of 229E; Also is immunomodulatory and cytoprotective effects, inhibition of the interaction between bacteria and host cells; Inhibits viral hemagglutination and Neuraminidase (NA) activity	[52] [53] [54] [55]
*Psidium guajava* CN: Guava	Eugenol (an)	SARS-CoV/Good binding affinity with Mpro and S protein	[34] [21]
*Scutellaria baicalensis* Georgi. CN: Skullcap	Baicalin (g) and scutellarein (l)	SARS-CoV/Inhibits nsP13 in vitro by affecting ATPase activity	[56] [57] [46]
*Thuja orientalis* L. CN: Tree of life	Essential oils: b-ocimene, 1,8-cineole, a-pinene and b-pinene mainly (ad)	SARS-CoV, HSV-1/Inhibitory activity against viral replication in vitro by visually scoring of the virus-induced cytopathogenic effect post-infection	[58] [29]
*Laurus nobilis* L. CN: Laurel			
*Salvia officinalis* L. CN: Sage			
*Urtica dioica* L. CN: Nettle	Lectin agglutinin (w)	SARS-CoV/Inhibits the end of the virus cycle infection	[29] [47]
*Polygonum cuspidatum* L. CN: Japanese knotty grass	Anthraquinone derived emodin (a)	SARS-CoV, HCoV-OC43/inhibits by blocking viral entry by binding to the S protein and interfering with the 3CLpro activity of the SARS-CoV and prevented the formation of the Nsp required for viral replication; Blocked the interaction between SARS-CoV S protein and ACE2, inhibited ion channel 3a and interrupted the release of new coronaviruses	[59] [28]
*Senna obtusifolia* L. CN: Abejorra	Emodin (a)		
*Rheum* spp. CN: Rhubarb			
*Aloe* spp. CN: Aloe	Aloe emodin (a)		[27]
*Vaccinium* spp. CN: Blueberry, mortiño, Agráz, among others.	Anthocyanins (t), myricetin (n), gallic acid (u), stilbenoid resveratrol (s) and procyanidins (ai)	SARS-CoV, MERS-CoV/(t) inhibits the production of NO and the secretion of TNF-α in macrophages induced by LPS-INF-γ caused by protocatechic acid, also show ACE inhibitory activity; (n) inhibits the coronavirus helicase protein by affecting the ATPase activity in vitro; Gallic acid decreases the secretion of MCP-1, ICAM-1, and VCAM-1 in endothelial cells; (s) partially mitigates induced cell death and reduces infectious viral replication; (v) possibly blocks the entry of cells through endocytosis	[60] [61] [62] [63]
*Vitis vinifera* L. CN: Red grape			
*Zingiber officinale* Rosc. CN: Ginger	[6]-gingerol (ak)	SARS-CoV-2/TMPRSS2 receptor blocking	[64] [65]

**Table 3:** Studies based on food for human consumption ACEi activity () and inhibition of AII to AT1R binding activity (ATRi). Individual results are given (ACE-%; ATR-%), based on studies by Patten et al., (2012) y Patten et al., (2016) [38,60].

Family	Common Name of Plant with ACE and AT1R Inhibition Activities (%, %)
Actinidiaceae	Gold kiwi (−0.2; 20.5), green kiwi (16.6; 2.5)
Agaricaceae	Button mushroom (12.5; 0.3)
Alliaceae	Chives (23.2; 28.4), garlic (6.8; 27.4), leek (2.8; 42.7), onion (−1.2; 34.2), shallot (0.9; 11.5), red onion (−4.0; 31.8), spring onion (6.4; 53.3), white onion (−1.2; 18.8)
Amaranthaceae	Spinach (−0.7; 29.6)
Apiaceae	Black carrot juice (91.1; 31.0), carrot (0.7; 5.0), coriander leaf (37.4; 56.6), coriander seed (11.7; 16.4), fennel (−2.1; 15.2), parsley (8.2; 41.3)
Arecaceae	Coconut (11.8; −18.0)
Asparagaceae	Asparagus (35.1; 27.7)
Asteraceae	Radicchio (56; 43.5), red coral lettuce (31.5; 15.8), tarragon (32.1; 30.7)
Auricularaceae	Wood Ear mushroom (13.1; 33.4)
Betulaceae	Hazelnut (−9.8; 25.1)
Brassicaceae	Bok choi (7.1; 30.4), broccoli (6.1; 0.2), brussel sprout (10.3; 1.2), Chinese broccoli (21.9; 38.7), Chinese cabbage (6.5; 28.8), choi sum (21.8; 2.6), red cabbage (24.6; 6.0), savoy cabbage (2.2; 52.1), watercress (18.7; 27.9), yellow mustard seed (5.2; −1.8)
Chenopodiacea	Silver beet (−1.0; 31.7), rainbow silver beet (−3.2; 10.2), beetroot (0.8; 6.2)
Combretaceae	Kakadu plum (48.7; 0.0)
Convolvulaceae	Red sweet potato (8.6; 16.5), sweet potato (4.9; 26.0)
Cucurbitaceae	Choko (5.2; 3.4), choko skin (53.2; 14.0), cucumber (14.6; 40.8), pumpkin (3.3; 1.1), squash (4.3; 46.0), zucchini (16.0; 11.8)
Ericaceae	Blueberry (−0.1; 43.3)
Fabaceae	Green bean (10.7; 27.2), green pea (−7.2; 9.3), lupin (−15.4; 12.1), Parafield lupin (−24.3; 7.6), peanut (1.4; −16.7)
Fagaceae	Chestnut (61.7; −5.6)
Juglandaceae	Pecan nut (0; 7.8), walnut (−10.9; 2.4)
Lamiaceae	Green basil (37.9; 26.4), purple basil (46.3; 11.0), Thai basil (69.5; 36.5), oregano (67.5; 55.7), rosemary (91.0; 55.7), sage (89.3; 68.2), thyme (87.4; 42.1)
Lauraceae	Avocado (6.2; 43.4), bay leaf (34.9; 37.3), cinnamon (100.0; 54.4), Indian bay leaf (28.7; 0.4)
Lythraceae	Pomegranate flesh (−6.2; 10.7)
Marasmiaceae	Enoki mushroom (4.8; −3.7), Shiitake mushroom (26.4; 11.8)
Meriplaceae	Maitake mushroom (67.0; 32.1)
Myrtaceae	Clove (66.1; 30.8), cedar Bay cherry (63.8; 2.1), riberry (11.3; −12.1)
Poaceae	Corn (0; 27.8), lemongrass (5.0; 7.2)
Podacarpaceae	Illawarra plum (100; 7.0)
Polygonaceae	Rhubarb (16.3; 8.5)
Rosaceae	Quince (12.3; 11.1), raspberry (6.2; 6.2), strawberry (20.3; 3.5), red delicious apple (6.8; 1.5)
Rubiaceae	Columbian dark coffee bean (63.416.0), Mocha coffee bean (56.7; 21.5)
Rutaceae	Desert lemon (6.1; −0.6), green finger lime (11.5; 15.8), red finger lime (−6.3; 13.7), green citrus (14.8; 21.1), lemon skin (12.4; 7.9), lime (−16.4; 6.2), lime skin (47.1; 33.8), mandarin (0.2; 3.6), navel oranges (6.5; −3.9), orange skin (46.1; 7.8), red citrus (2.9; 40.1), red citrus skin (11.8; 17.4), ruby grapefruit (6.6; 14.9), Valencia orange (1.5; 5.4), yellow citrus (5.1; 18.6), yellow citrus skin (10.5; 7.3)
Saccharomycetaceae	Brewer’s yeast (31.8; −19.3)
Santalum	Quandong (40.6; 8.5)
Solanaceae	Potato (1.6; 16.6)
Sterculiaceae	Cocoa bean (81.2; 10.5)
Theaceae	English breakfast black tea (88.8; 27.1), green tea (41.1; 12.4), Japanese green tea (100; 41.6), Madura black tea (100; 30.5)
Pleurotaceae	Oyster mushroom (35.9; 16.1), Honey Brown mushroom (14.6; 8.6)
Vitaceae	Muscat grape (59.0; −2.8), white grape seed (100; 0.0), red grape skin (92.7; 14.4), Chambourcin grape (58.2; 10.6), Muscat Hamburg grape (73.5; -7.9), Cabinet Sauvignon grape (72.3; 0.0), Sun Muscat grape (59.0; −1.0), Concord grape (49.3; −3.3),
Zingiberaceae	Cardamom (7.4; 1.2), ginger (9.9; 38.0), tumeric (15.1; −1.4)

**Table 4:** Plant species with potential for the bioprospecting of secondary metabolites located in Ecuador.

Family	Potential Species	Origin	Region	Potential Anti-Sars Effect	References
Betulaceae	Birches (*Betula* spp.)	Introduced	Sierra region	Anticoagulants and antirheumatic	[69]
Burseraceae	Palo santo (*Bursera graveolens* Triana and Planch)	Native	Coast and Sierra regions	Anti-inflammatory and antioxidant	[70]
Ericaceae	Mortiño (*Vaccinium floribundum* Kunth)	Endemic	Sierra region	Antioxidant	[29]
Euphorbiaceae	Croto de monte (*Croton rivinifolius* Kunth)	Endemic	Coast region	Anticarcinogenic and antiviral	[70]
	Dog tongue (*Euphorbia neriifolia* L.)	Introduced	Coast región	Antitussive, antifungal and antitumor	[69]
Fabaceae	Frijolillo (*Cassia tora* L.)	Native	Coast región	Anticoagulants and anti-inflammatory	[70]
	White rain (*Gliricidia brenningii* Harms)	Native	Coast región	Antiherpetic and anticarcinogenic	[29]
Orchidaceae	Orchid (*Dendrobium* spp.)	Introduced	Coast, Sierra y Amazon regions	Antiviral	[71]
	Guayaquil Orchid (*Encyclia angustiloba* Schltr)	Endemic	Coast región	Antiviral	[71]
Polygonaceae	Bloodroot (*Polygonum arenastrum* Boreau)	Introduced	Coast y Amazon regions	Antiviral	[59]
Rubiaceae	Cascarilla (*Cinchona pubescens* Vahl)	Native	Sierra region	Febrifuge, antiviral	[72]
	Cat’s claw (*Uncaria tomentosa* D. C.)	Native	Sierra and Amazon regions	Anti-inflammatory	[72]
	Colorado (*Simira ecuadoriensis* Standl)	Endemic	Coast region	Febrifuge and antiviral	[72]
	Crucita (*Rosenbergiodendron formosum* Fagerl.)	Native	Coast region	Febrifuge and antiviral	[72]
Scrophulariaceae	Escrofularia (*Scrophularia* spp.)	Introduced	Coast región	Anti-inflammatory and antimicrobial	[69]
Urticaceae	Nettle (*Urtica urens* L.)	Introduced	Sierra region	Antiviral	[72]

## Data Availability

Not applicable.
